# High dose isoleucine stabilizes nuclear PTEN to suppress the proliferation of lung cancer

**DOI:** 10.1007/s12672-023-00634-1

**Published:** 2023-02-23

**Authors:** Haiqing Wang, Sen Chen, Wenhui Kang, Bojiao Ding, Shulan Cui, Li Zhou, Na Zhang, Huiying Luo, Mingjuan Wang, Fan Zhang, Zezhou Zhao, Zihu Guo, Chao Wang, Liang Li, Zhengzhong Wang, Xuetong Chen, Yonghua Wang

**Affiliations:** 1 Key Laboratory of Resource Biology and Biotechnology in Western China, Ministry of Education, School of Life Sciences, Northwest University, No. 229 Taibai North Road, Xi’an, 710069 Shaanxi China; 2 State Key Laboratory of New-Tech for Chinese Medicine Pharmaceutical Process, Jiangsu Kanion Parmaceutical Co. Ltd., Lianyungang, 222002 Jiangsu China; 3 College of Pharmacy, Heze University, Heze, 274015 Shandong China; 4 Immune Cells and Antibody Engineering Research Center in University of Guizhou Province, Key Laboratory of Biology and Medical Engineering, School of Biology and Engineering (School of Modern Industry for Health and Medicine), Guizhou Medical University, Guiyang, 550025 China; 5 Collaborative Innovation Center of Qiyao in Mt. Qinling, Yangling, 712100 Shaanxi China; 6 School of Traditional Chinese Medicine, Baoji Vocational Technology College, Baoji, 721000 Shaanxi China

**Keywords:** Branched-chain amino acids, Isoleucine, Tumor therapy, PTEN, IARS

## Abstract

**Purpose:**

Cancer cells require a supply of amino acids, particularly essential amino acids such as branched-chain amino acids (BCAAs, i.e., valine, leucine, and isoleucine), to meet the increased nutrient demands of malignant tumors. The cell-autonomous and non-autonomous roles of altered BCAA supply have been implicated in cancer progression. The critical proteins involved in BCAA uptake, transport, metabolism, etc. serve as potential therapeutic biomarkers in human cancers. Here, we summarize the potential anti-tumor mechanism of BCAA by exploring the chain reaction triggered by increased BCAA supply in the tumor.

**Method:**

A system-wide strategy was employed to provide a generic solution to establish the links between BCAA and cancer based on comprehensive omics, molecular experimentation, and data analysis.

**Results:**

BCAA over-supplementation (900 mg/kg) significantly inhibited tumor growth and reduced tumor burden, with isoleucine having the most pronounced effect. Surprisingly, isoleucine inhibited tumor growth independently of mTORC1 activation, a classical amino acid sensor. Exploratory transcriptome analysis revealed that Phosphatase and tensin homolog (PTEN) is the critical factor in the anti-tumor effect of isoleucine. By inhibiting PTEN ubiquitination, isoleucine can promote PTEN nuclear import and maintain PTEN nuclear stability. Interestingly, this process was regulated by isoleucine-tRNA ligase, cytoplasmic (IARS), a direct target of isoleucine. We demonstrated the enhanced interaction between IARS and PTEN in the presence of excess isoleucine. At the same time, IARS knockout leads to loss of isoleucine tumor suppressor ability.

**Conclusion:**

Overall, our results provide insights into the regulation of the IARS-PTEN anti-tumor axis by isoleucine and reveal a unique therapeutic approach based on enhancing cellular isoleucine supply.

**Supplementary Information:**

The online version contains supplementary material available at 10.1007/s12672-023-00634-1.

## Introduction

Amino acid requirements are a hallmark of tumor progression. Metabolic reprogramming dramatically enhances the ability of cancer cells to capture amino acids to support the abnormal proliferation and migration of tumors [1]. Surprisingly, the fluctuating supply of amino acids could disrupt the amino acid balance in the TME, affecting the occurrence and development of tumors [2–4]. However, based on this strategy, the new therapies face a difficult choice: deficiency or excess. Supplementation or depletion of the 20 standard amino acids has significantly different effects in various stages of tumorigenesis and development [5]. Targeting the intake, absorption, transport, synthesis, and consumption of amino acids, including glutamine [6], asparagine [7], arginine [8], methionine [9], and cysteine [10], etc., can play a significant role in the treatment of tumors. Despite these important advances, it is necessary to implement this treatment strategy at different stages of cancer and optimize the amino acid dosage range.

Among these amino acids, branched-chain amino acids (BCAA, including valine, leucine, and isoleucine) are essential amino acids that regulate tumor growth and proliferation [11–13]. Accordingly, exogenous BCAA can be explored as a “drug” and reduce endogenous amino acid interference. Previous studies have shown conflicting effects on different types of tumors with BCAA deprivation and supplementation [13–15]. Currently, leucine transport inhibition blocks ER + breast cancer growth [16], while BCAA supplementation may suppress the development and metastasis of breast cancer [17]. Furthermore, leucine, but not isoleucine or valine, was associated with obesity-related cancer and cancer mortality [18]. These studies suggest that the complex mechanism of isoleucine, leucine, or valine in signaling, and metabolic pathways is responsible for the differential utilization of BCAAs in tumors. However, the fewer and more contradictory clinical research data arouse our curiosity about the underlying mechanism of BCAA tumor therapy.

Lung cancer, the first leading cause of cancer-related death in China, is a significant health burden for unfortunate patients [19]. Non-small cell lung cancer (NSCLC) is common cancer with a high incidence. Recent genetic profiling has shown that NSCLC has a preponderance of mutations affecting PTEN [20], the SETD1A/Wnt/β-catenin feedback loop [21], and the AKT-mTOR pathway [22]. However, targeting them effectively for preventive or therapeutic interventions has proven difficult and largely unsuccessful. Therefore, modulating the supply of BCAAs to stop tumor growth may be a promising therapeutic strategy for the treatment of NSCLC. To determine the uncertain effect of BCAAs on lung cancer, we have used unbiased, comprehensive transcriptomic analyses and a variety of experimental approaches to characterize NSCLC and BCAAs.

## Material and methods

### Key chemicals and reagents

L-Isoleucine (Cat No. S20042, 99%), L-Leucine (Cat No. S20044, 99%) and L-Valine (Cat No. S20117, 99%) were purchased from Shanghai Yuanye Technology Co. Fetal Bovine Serum (Cat No. 10099141), Taipan Blue (Cat No. T10282), Dulbecco’s Modified Eagle’s Medium (DMEM, Gibco) and RPMI-1640 (Gibco) were purchased from Thermo Fisher Scientific Co. Ltd.; Penicillin mixture (Cat No. P1400) was purchased from Beijing Solabao Technology Co. Ltd.; Ampicillin (Cat No. A1170) was purchased from Beijing Solabao Technology Co. Ltd.; Rapamycin (Cat No. B20714) was purchased from Shanghai Yuanye Technology Co.; Lipofectamine 2000 (Cat No. 11668019) and Pierce^™^ ECL Western Blotting Substrate (Cat No. 32209) purchased from Thermo Fisher Scientific, Inc.; EnGen^®^ Sau Cas9 nuclease (Cat No. M0654S) purchased from NEB; Polybrene (Cat No. TR-1003-G) purchased from Sigma-Aldrich.

### Cell lines and culture

Human NSCLC cell lines (NCI-H1975 and A549) and mouse Lewis lung carcinoma (LLC) cell lines were purchased from the Chinese Academy of Sciences Shanghai cell bank (Shanghai, China). Mouse colon cancer cells CT26 and mouse melanoma cells B16F10 were from Jiangsu Kanion Modern TCM Research Institute. Mouse breast cancer cells 4T1 and liver cancer cells Hepa1-6 were purchased from Saiye (Guangzhou) Biological Technology Co., Ltd. Human renal clear cells adenocarcinoma 786-O was purchased from Procell Life Science & Technology Co., Ltd. A549 and LCC cells knocked out PTEN and A549 cells knocked down PTEN were constructed by in house. 293 T cells were obtained from the State Key Laboratory of New-tech for Chinese Medicine Pharmaceutical Process.

A549, LLC, Hepa1-6, and B16F10 cells were cultured using DMEM medium supplemented with 10% FBS (Gibco, USA). CT26, H1975, 786-O, and 4T1 cells were incubated with RPMI-1640 medium (Gibco, Life Technologies, California, USA) supplemented with 10% FBS. Based on previous studies, rapamycin at a concentration of 50 nM was used in tumor cell proliferation assays [23]. All cells were cultured in a humidified cell incubator at 37 ℃, 5% CO2.

### Cell proliferation experiment

Cell Proliferation was assessed using Cell Counting Kit-8 (CCK-8) (Shanghai, China). Briefly, cells were seeded in 96-well plates at a concentration of 3 × 10^3^ per well. After culturing for 2, 4, and 6 days, CCK-8 solution (10 µl) was added to each well at the indicated time points. Plates were then stored at 37 ℃ for 2 h, and the number of viable cells was estimated by measuring absorbance at 450 nm and 600 nm. All experimental results were repeated three or more times.

For cells in DMEM medium, the specific dose of BCAA is 25× (Lsoleucine 2.625 g/L, Leucine 2.625 g/L, Valine 2.35 g/L); 50× (Lsoleucine 5.25 g/L, Leucine 5.25 g/L, Valine 4.7 g/L); 75 × (Lsoleucine 7.875 g/L, Leucine 7.875 g/L, Valine 7.05 g/L); cells in RPMI 1640 medium, the specific dose of BCAA is 25× (Lsoleucine 1.25 g/ L, Leucine 1.25 g/L, Valine 0.5 g/L); 50 × (Lsoleucine 2.5 g/L, Leucine 2.5 g/L, Valine 1 g/L); 75× (Lsoleucine 3.75 g/L, Leucine 3.75 g/L, Valine 1.5 g/L).

### Quantitative real-time PCR (RT-qPCR)

Total RNA was extracted with TaKaRa MiniBEST Universal RNA Extraction Kit (Takara, Japan), and concentration was measured by spectrophotometer. Reverse transcription was performed to synthesize cDNA using PrimeScript^™^ RT reagent Kit with gDNA Eraser (Takara, Japan). The reaction was performed in StepOnePlus Real-Time PCR System (Thermo Fisher Scientific) with the mixture of cDNA, primers, TB Green^®^ Premix Ex Taq^™^ (Tli RNaseH Plus), ROX plus (Takara, Japan). The primer sequences were shown in Table 1. Gene expression levels were measured using the Step One Plus Real-Time PCR System (Applied Biosystems, USA), using Gapdh as the internal control. Data were analyzed by the 2^−ΔΔCt^ method. The results from 3 independent repeat assays, performed in different plates, each using different cDNAs from the cultures analyzed, were averaged to produce a single mean quantity value for each mRNA.

**Table 1:** The primer sequences information

Gene	Forward primers	Reverse primers
PTEN	TGAGTTCCCTCAGCCGTTACCT	GAGGTTTCCTCTGGTCCTGGTA
*Pten*	TGAGTTCCCTCAGCCATTGCCT	GAGGTTTCCTCTGGTCCTGGTA
GAPDH	ACAACTTTGGTATCGTGGAAGG	GCCATCACGCCACAGTTTC
*Gapdh*	AGGTCGGTGTGAACGGATTTG	TGTAGACCATGTAGTTGAGGTC
*Iars1*	CTCCTTGGCTTCTTTGAGACCG	CCTCCTGTTCATTCGGACATACC

### Western blotting

Cells or tissues were collected and lysed using the QProteome^™^ Mammalian Protein Prep Kit (Qiagen, Hilden, Germany). Nuclear and cytoplasmic proteins were extracted and isolated using the NE-PER Nuclear and Cytoplasmic Extraction Reagents kit (Thermo Fisher Scientific Inc.). After centrifugation at 16,000 × g for 10 min, the protein concentration in the supernatant was determined using the BCA protein assay kit (Bio-Rad, Shanghai, China). Equal amounts of protein were boiled by adding 4 × Sample Loading Buffers for 10 min at 100 ℃ and resolved using SDS-PAGE. Information for antibodies is as follows: Phospho-p70 S6 Kinase (Thr389) (9234), p70 S6 Kinase Antibody (9202), PTEN (D4.3) XP Rabbit mAb (9188), Ubiquitin Antibody (3933), Histone H3 Antibody (9715), and Anti-FLAG tag Antibody (14793) were purchased from Cell Signaling Technology (Danvers, MA, USA). PTEN Polyclonal Antibody (22034-1-AP), IARS Polyclonal Antibody (26942-1-AP), Anti-IARS2 Antibody (17170-1-AP), WWP2 Polyclonal Antibody (12197-1-AP), USP7 Polyclonal Antibody (26948-1-AP), USP13 Polyclonal Antibody (16840-1-AP), FBXO22 Polyclonal Antibody (13606-1-AP) were purchased from Proteintech (Rosemont, IL, USA). Anti-IARS Antibody (ab31533), Rabbit Anti-Mouse IgG H&L (ab6728), Anti-GAPDH Antibody (ab181602), Anti-WWP1 Antibody (ab43791), Anti-Lamin B1 antibody (ab16048), Anti-Cdk4 antibody (ab199728), Anti-DMRT1 antibody (ab222895) were purchased from Abcam (Cambridge, MA, USA). PTEN Monoclonal Antibody (17.A) (MA5-12278), alpha Tubulin (PA5-16891) were purchased from Thermofisher. Anti-Nedd4 Antibody was purchased from MilliporeSigma. All experimental results were repeated three or more times.

### RNAseq data analysis

The cDNA/DNA/Small RNA libraries were sequenced on the Illumina sequencing platform by Genedenovo Biotechnology Co., Ltd (Guangzhou, China). Bioinformatics analysis of the reads, including the filtering of clean reads, the alignment with ribosome RNA (rRNA) and the reference genome, and the quantification of gene abundance, was performed according to the default parameters [24]. Then, RNAs differential expression analysis was performed by DESeq2 [25] software between the control group and the isoleucine treatment group. Genes/transcripts with p-value < 0.05 and the absolute value of log2 (Fold change) >  = 1 are differentially expressed genes/transcripts (DEGs). The original contributions presented in the study are publicly available. This data can be found here: https://www.ncbi.nlm.nih.gov/geo/query/acc.cgi?acc=GSE220544.

Based on the RNAseq data of clinical samples of NSCLC in the TCGA database, the correlation analysis of IARS1, IARS2, and PTEN expression was analyzed using the GEPIA2 tool [26].

### Plasmid DNA extraction and transfection

The plasmid DNA was extracted by the Endo-Free Plasmid Maxi Kit (Cat. No.: D6926). Transfection of plasmid DNA was performed using the Xfect^™^ Transfection Reagent Kit (Cat. No.: 631317). The day before transfection, about 3 × 10^5^ cells were seeded in a 6-well plate, and the complete medium was normally cultured so that the cells were 50 to 70% confluent at the time of transfection; vortex Xfect Polymer thoroughly; in a centrifuge tube, first add 5 µg of plasmid DNA, then add Xfect Reaction Buffer solution to make the final volume 100 µL, then vortex at high speed for 5 s to mix the plasmid DNA; next, add 1.5 μL of Xfect Polymer to the centrifuge tube, then vortex at high speed for 10 s to mix well, and then incubate at room temperature for 10 min; add the entire 100 μL nanoparticle complex solution dropwise to the cell culture medium and shake the 6-well plate gently back and forth to mix; incubate at 37 ℃ for 4 h, carefully remove the nanoparticle complexes from the cells, replace with 2 mL of fresh complete growth medium, and return the plate to the 37 ℃ incubator until analysis time. The plasmid information involved in this study is listed in Table 2. For plasmid transfection, Lipofectamine 2000 (Invitrogen, Waltham, MA, USA) was used.

**Table 2:** The plasmid information

Plasmid vector	Provider names	Vector ID
pLV[Exp]-Puro-CMV > FLAG/hPTEN[NM_001304717.5]*/EGFP	VectorBuilder	VB201207-1204wbw
pLV[Exp]-Puro-CMV > FLAG/hIARS1[NM_001378569.1]/EGFP	VectorBuilder	VB200810-1690bab
pRP[Exp]-EGFP/Puro-CAG > hPTEN[NM_001304717.5]	VectorBuilder	VB900005-2059vze
pLV[shRNA]-EGFP:T2A:Puro-U6 > hIARS[shRNA#2]	VectorBuilder	VB900047-2368gnu
pLV[shRNA]-EGFP:T2A:Puro-U6 > hPTEN[shRNA#1]	VectorBuilder	VB900058-2697amv
pLV[Exp]-EGFP:T2A:Puro-CMV > FLAG/hPTEN[NM_001304717.5]*	VectorBuilder	VB210506-1296xka
pRP[Exp]-Puro-CMV > hPTEN[NM_000314.8]/EGFP	VectorBuilder	VB210408-1024xgy
pRP[Exp]-Puro-CMV > {hPTEN[NM_000314.8]*(K13E,K289E)}/EGFP	VectorBuilder	VB210408-1026qmu
pLV[shRNA]-EGFP:T2A:Puro-U6 > hIARS[shRNA#2]	VectorBuilder	VB900047-2368gnu
pLV[shRNA]-EGFP:T2A:Puro-U6 > Scramble_shRNA#1	VectorBuilder	VB010000-0009mxc
pCMV-VSV-G	Addgene	8454
pCMV-dR8.2 dvpr	Addgene	8455

### Lentiviral packaging

The lentiviral plasmid, pCMV-Δ8.2 and pCMV-VSVG plasmids were co-transfected into 293 T cells, and the medium was changed to fresh medium 4 h after transfection and then cultured for 48 h. The collected supernatant medium was the virus medium. Next, filter through a 0.45 μm filter to remove cell debris, and store at − 80 ℃ or directly use to infect cells. For lentiviral packaging, the pCMV delta R8.2 (Addgene 12263) and pCMV-vsv-g (Addgene 8454) systems were utilized.

### Construction of knockdown or overexpression cell lines

About 3 × 10^5^ A549 cells were inoculated in a 6-well plate, and the complete medium was cultured normally. After the cells were completely attached to the wall, the medium was aspirated, and 1 mL of fresh medium and 1 mL of virus liquid were added. Add Polybrene at a ratio of 1:1000, infect for 24 to 48 h, and detect the transfection efficiency with a fluorescence microscope. If green fluorescence was observed, fresh medium was replaced and puromycin was added for screening. Cells with only green fluorescence were monoclonal obtained by the limiting dilution method. After expansion and culture, the cells were collected for qPCR or Western Blot identification, and monoclonal cells with normal identification results were selected for cryopreservation.

### Cell construction for knocking out PTEN

The online biological information website CRISPOR (tefor.net) is used to analyze the appropriate sgRNA. Then the cell targeting site is sequenced and tested, and the gRNA single strand is synthesized after passing the test. The human PTEN gene gRNA sequence: 5′-AAAAGGATATTGTGCAACTG-TGG-3′, the mouse *Pten* gene gRNA sequence is: gRNA-A1: AGGCCTGGGTGACGTGCATT-TGG; gRNA-A2: GGGATGAGGGATACACTAAG-TGG; gRNA-B1: GCTGTAGTAATATCTGCTAT-TGG; gRNA-B2: CACCTACTCCAGGTAGGTCT-TGG, wherein TGG is a PAM sequence. The Cas9 protein and sgRNA single strand were transfected into A549 or LLC cells by electroporation. Using the limited dilution method and the PCR amplification-sequencing method, a cell population is cultured and screened with only a single clone, and then the screened single clone is expanded and cultured (Supplementary Fig. 1). Finally, a human PTEN gene-knockout A-549 cell line and a mouse *Pten* gene-knockout LLC cell line were obtained.

### Immunofluorescence

Cells were fixed with 4% paraformaldehyde for 10 min; then washed 3 times with PBS, 5 min each; permeabilized with 0.25% Triton X-100 for 10 min; blocked with 1% BSA for 1 h; then washed 3 times with PBS, 5 min each; Incubate with primary antibody overnight at 4 ℃; recover primary antibody and wash with PBS 3 times, 5 min each; add secondary antibody and incubate at room temperature for 1 h; then wash with PBS 3 times, 5 min each; finally add DAPI staining solution and incubate at room temperature for 10 min, and photographed using a fluorescence microscope. All experimental results were repeated three or more times.

### Immunofluorescence colocalization

For the cells in the black 96-well plate, remove the medium carefully, add 50 μL of 4% paraformaldehyde, and fix for 10 min; then use PBS solution to wash 3 times, each time for 5 min; then add 50 μL of 0.25% Triton X-100, permeate for 10 min; remove the liquid, then add 50 μL of 1% BSA to block for 1 h; then wash with PBS 3 times, each time for 5 min; incubate overnight at 4 ℃ with rabbit anti-IARS1 and mouse anti-PTEN primary antibody; recover the primary antibody; wash with PBS 3 times, 5 min each time; add anti-mouse and anti-rabbit secondary antibodies and incubate at room temperature in the dark for 1 h; recover the secondary antibody, add 100 μL PBS to wash 3 times, 5 min each time; add 30 μL DAPI staining solution, and incubate at room temperature in the dark for 10 min; finally use a fluorescence microscope to examine and take pictures. All experimental results were repeated three or more times.

### Bioinformatics analysis

The DEGs were converted into human homologous genes using the R package biomaRt [27]. Then the obtained homologous genes were analyzed for transcription factor enrichment using the KnockTF [28] to obtain potential factors regulating these DEGs. Enrichment analysis of Gene Ontology Biological Processes (GOBP) was performed using Metascape [29] and ClueGO [30].

### Animal experiment

All animal protocols described in this study were approved by the Institutional Animal Care and Use Committee (IACUC: 2021091001, 2021112502) at the Kanion Parmaceutical. 5 × 10^5^ cells collected in serum-free medium were inoculated in the forelimb axilla of 6–8 week-old C57BL/6 male mice (LLC and B16F10 cells) or BALB/c female mice (4T1 and CT26 cells) (purchased from the Comparative medicine center of Yangzhou University and SPF (Beijing) Biotechnology Co., Ltd.). After inoculation, 900 mg/kg of BCAA (the ratio of isoleucine, leucine, and valine was 1:1:1) was injected intraperitoneally every day from the third day. For the control group, the same volume of PBS was injected. The length and width of the mouse tumors were measured every 2 days to see if the tumor size of the mice reached ethical limits. After ethical approval, the tumors were weighed and photographed. For isoleucine, leucine and valine, each group was given 300 mg/kg. In the pilot experiment, we have measured the food intake of the mice and found no significant difference.

To better confirm that the effect of isoleucine on inhibiting tumor growth is not related to the activation of mTOR signaling pathway, C57BL/6 male mice were inoculated with non-small cell lung cancer (LLC) and human NSCLC (H1975) according to the same method. From the third day, the intraperitoneal injection of isoleucine (300 mg/kg), rapamycin (1 mg/kg), or a combination of the two was administered.

The IACUC protocol in this study follows the guidelines for the ethical review of laboratory animal welfare People's Republic of China National Standard GB/T 35892–2018 [31]. The maximal tumor size/burden was not exceeded the permissible tolerance in IACUC protocol (< 2 cm).

### Co-immunoprecipitation and Nano-LC–ESI–MS/MS analysis

To confirm the potential interaction between IARS1/IARS2 and PTEN, protein A immunomagnetic bead immunoprecipitation kit was used for co-immunoprecipitation experiments. The obtained immunoprecipitated samples were analyzed by Western blot and sent for Nano-LC-ESI-MS/MS analysis to Bio-Tech Pack Technology (Beijing, China), respectively.

### Statistical analysis

The Welch’s t test was used to measure the correlation between two groups of variables. The Ordinary one-way ANOVA was used to compare variables in more than two groups. The significance level was set at *p* < 0.05, and all statistical tests were two-sided. All statistical data were analyzed using R software or online analysis tools described in the relevant Materials and Methods subsections.

## Results

### Excessive BCAA supplementation suppresses but not promotes tumor growth

**Fig. 1:**
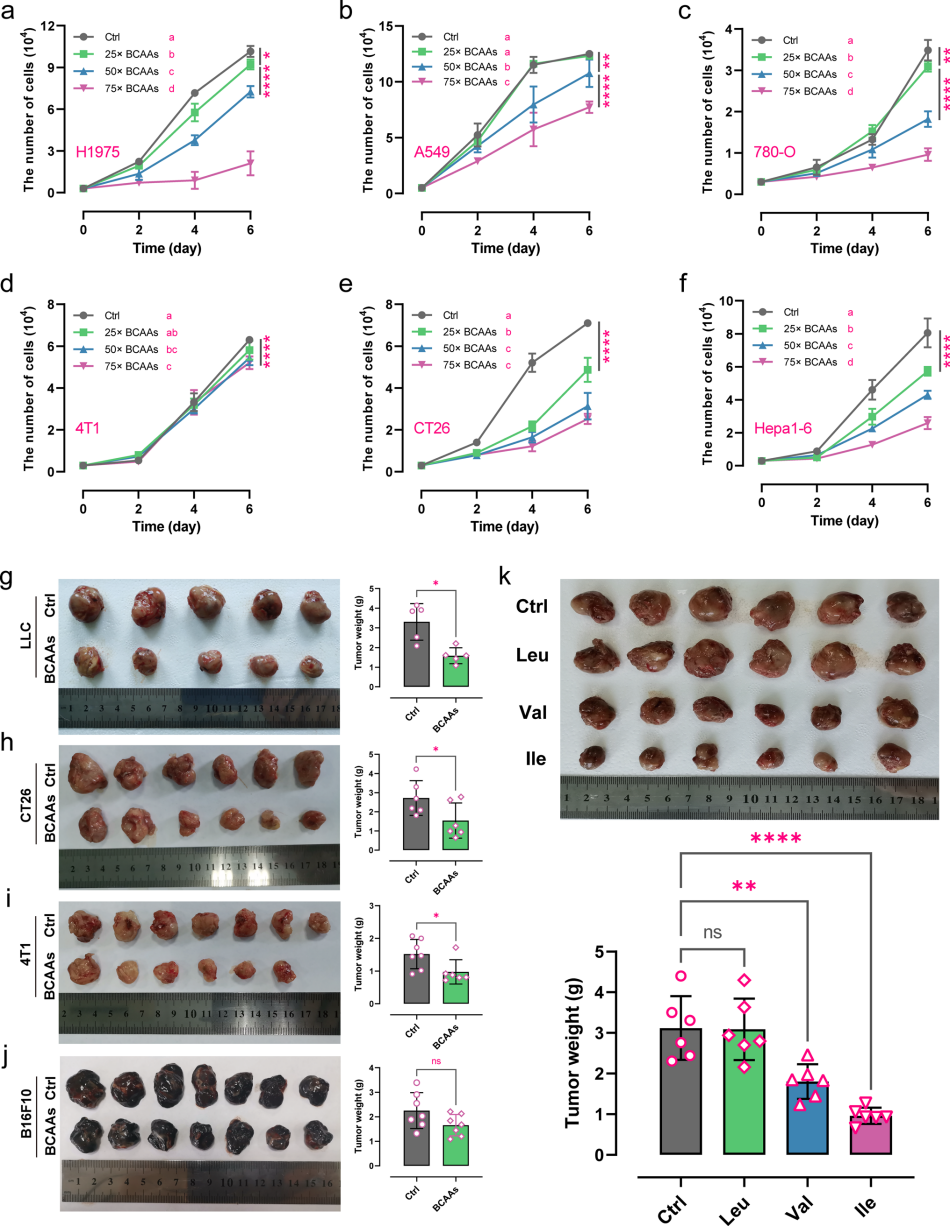
The effect of excess BCAA on tumor growth in vitro and in vivo. **a**–**f** Proliferation curves of H1975 **a**, A549 **b**, 786-O **c**, 4T1 **d**, CT26 **e**, and Hepa1-6 **f** cells cultured in medium containing different concentrations of BCAA (n = 6); **g**–**j** Assessment (tumor images and tumor weight) of BCAA on tumor growth based on LLC (n = 5), CT26 (n = 6), 4T1 (n = 7), and B16F10 (n = 7) tumor-bearing mice; **k** Assessment (tumor images and tumor weight) of leucine (Leu), valine (Val), or isoleucine (Ile) on tumor growth based on LLC-bearing mice (n = 6),. *ns* no significant difference; * *p* < 0.05; ** *p* < 0.01; **** *p* < 0.0001. Different letters, *p* < 0.05, same letters, no significant difference

To determine whether branched-chain amino acid (BCAA) supplementation is beneficial or detrimental to the growth of tumors, we cultured several tumor cell lines, including non-small cell lung cancer (NSCLC, A549 & H1975), breast cancer (4T1), liver cancer (Hepa1-6), colon cancer (CT26) and clear cell renal cell carcinoma (786-O), in medium containing high concentrations of BCAA (25x, 50x, 75x, see detailed concentrations in the Methods) to monitor the corresponding changes in proliferation curves (Fig. 1a–f). The results showed that the proliferation of H1975, A549, and 786-O was inhibited in a concentration-dependent manner when the concentration of BCAA in the medium reached more than 50 times that of the standard medium (> = 50-fold BCAA) (Fig. 1a–c). In contrast, an appropriate increase in BCAA concentration affected their proliferation (Fig. 1a–c, e–f). Furthermore, increasing the concentration of BCAA did not necessarily increase its inhibitory effect in 4T1 cells (Fig. 1d). In contrast, CT26 and Hepa1-6 cells were more sensitive to changes in BCAA concentration and showed significant proliferation inhibition in a culture environment with more than 25-fold BCAA (Fig. 1e–f). These results suggest that the over-supplementation of BCAA in a growth environment, i.e., nutrient adequacy, does not necessarily favor cancer cell proliferation.

Next, mouse tumor xenograft models, including colon cancer (CT26), melanoma (B16-F10), breast cancer (4T1), and non-small cell lung cancer (LLC), were established to investigate the anti-tumor effects of BCAA in vivo. Consistent with the in vitro results, BCAA significantly inhibited the growth of NSCLC (Fig. 1g, P value = 0.0106), colon cancer (Fig. 1h, P value = 0.0498), and breast cancer (Fig. 1i, P value = 0.0358) and reduced the tumor weight compared to the control group. However, BCAA had no significant effect on melanoma growth (Fig. 1j, P value = 0.0928). These results indicated that excessive BCAA supplementation in this novel anti-tumor therapy deserves further investigation, although it may not be appropriate for all tumors.

Hence, we also need to consider the amino acids in BCAA: leucine, isoleucine, and valine, which may exert different anti-tumor activities due to intricate biological mechanisms. We then investigated the main contributors of the three amino acids in a mouse xenograft model of NSCLC, which was most significantly affected by BCAA. Interestingly, isoleucine and valine significantly inhibited tumor growth and reduced tumor weight (Fig. 1k). The isoleucine group showed the most pronounced effect. Although leucine was the isomer of isoleucine, it had no effect on tumor growth (Fig. 1k). These results indicated that the anti-tumor activity of BCAA mainly depended on isoleucine and valine, among which excessive supplementation of isoleucine resulted in the most apparent inhibition of tumor weight.

### Isoleucine inhibits tumor growth independent of mTORC1 activation

**Fig. 2:**
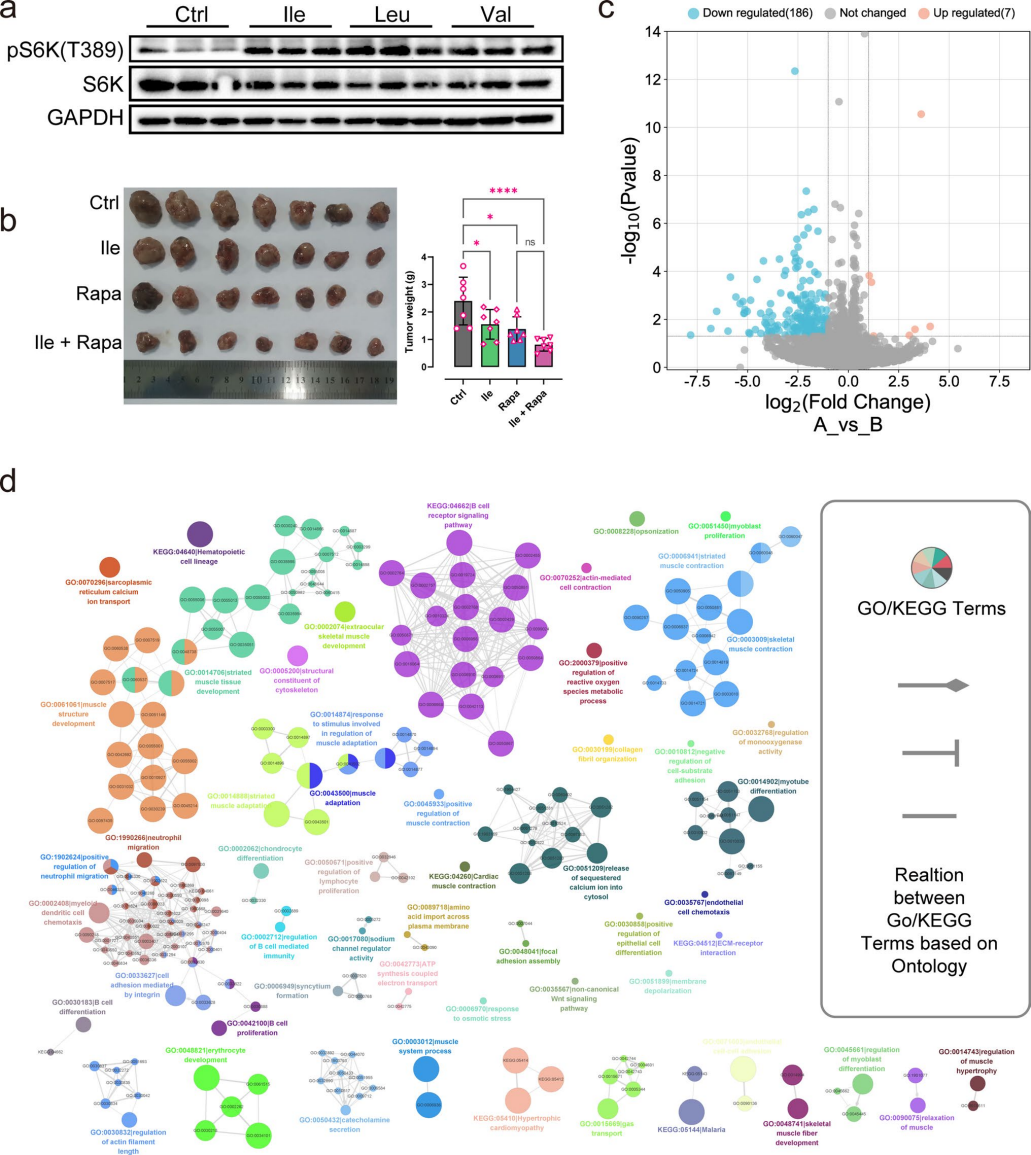
The preliminary study on the underlying mechanism of isoleucine's anti-tumor activity. **a** S6K phosphorylation level in LLC tumor tissues by Western blot analysis (n = 3); **b** Assessment (tumor images and tumor weight) of rapamycin (Rapa, 1 mg/kg) and isoleucine (Ile, 300 mg/kg) on tumor growth based on LLC-bearing mice (n = 7); **c** The volcano plot of transcriptome data (n = 3); **d** GOBP enrichment analysis of differentially expressed genes was performed using GlueGO. *ns* no significant difference; * *p* < 0.05; **** *p* < 0.0001

The mTORC1 signaling pathway is a classic pathway by which BCAA affects tumor development [32]. Therefore, the phosphorylation levels of Ribosomal Protein S6 Kinase B1 (RPS6K), an indicator of mTORC1 activation in response to amino acids, were determined to verify the underlying mechanism of excess isoleucine supplementation against tumors. Consistent with previous studies, the phosphorylation levels of RPS6K were significantly up-regulated in isoleucine, leucine, and valine-treated tumor tissues compared to the control group (Fig. 2a), indicating activation of the mTORC1 signaling pathway. However, activation of the mTORC1 pathway can promote anabolism and inhibit catabolism, thereby contributing to tumorigenesis [33]. Consistently, rapamycin (Rapa), an inhibitor of mTORC1, decreased the phosphorylation level of RPS6K and significantly inhibited the proliferation of H1975 cells (Supplementary Fig. 2a–b). Meanwhile, the combination of rapamycin and isoleucine did not restore the proliferation status of H1975 cells but enhanced the inhibitory effect of isoleucine (Supplementary Fig. 2a–b). Consistent with the in vitro findings, experiments in the mouse NSCLC tumor xenograft models showed that either isoleucine or rapamycin treatment alone significantly reduced tumor weight, whereas combination treatment did not restore tumor growth (Fig. 2b). Taken together, these data suggested that isoleucine inhibits tumor growth independently of mTORC1 signaling pathway.

To explore the potential mechanism of isoleucine anti-tumor, we performed transcriptome data analysis of isoleucine-treated LLC tumor tissues and obtained 193 differentially expressed genes (DEGs), of which 7 were up-regulated, and 186 were down-regulated (Fig. 2c & Supplementary Fig. 2c and supplementary Table 1). Gene Ontology Biological Process (GOBP) enrichment analysis based on multiple strategies revealed that the DEGs were associated with multiple biological processes, including GO:0035567-non-canonical Wnt signaling pathway, GO:0005200-structural constituent of cytoskeleton, KEGG:04512-ECM-receptor interaction, GO:0010812-negative regulation of cell-substrate adhesion, GO:0002712-regulation of B cell mediated immunity,GO:0089718-amino acid import across plasma membrane, GO:0048041-focal adhesion assembly, GO:1902624-positive regulation of neutrophil migration, GO:0002408-myeloid dendritic cell chemotaxis, etc. (Fig. 2d & Supplementary Fig. 1d and Supplementary Table 2). These results suggest that excessive supplementation of isoleucine could modulate a wide range of biological processes in the tumor microenvironment to inhibit tumor growth.

### PTEN is a critical factor associated with isoleucine’s anti-tumor activity

**Fig. 3:**
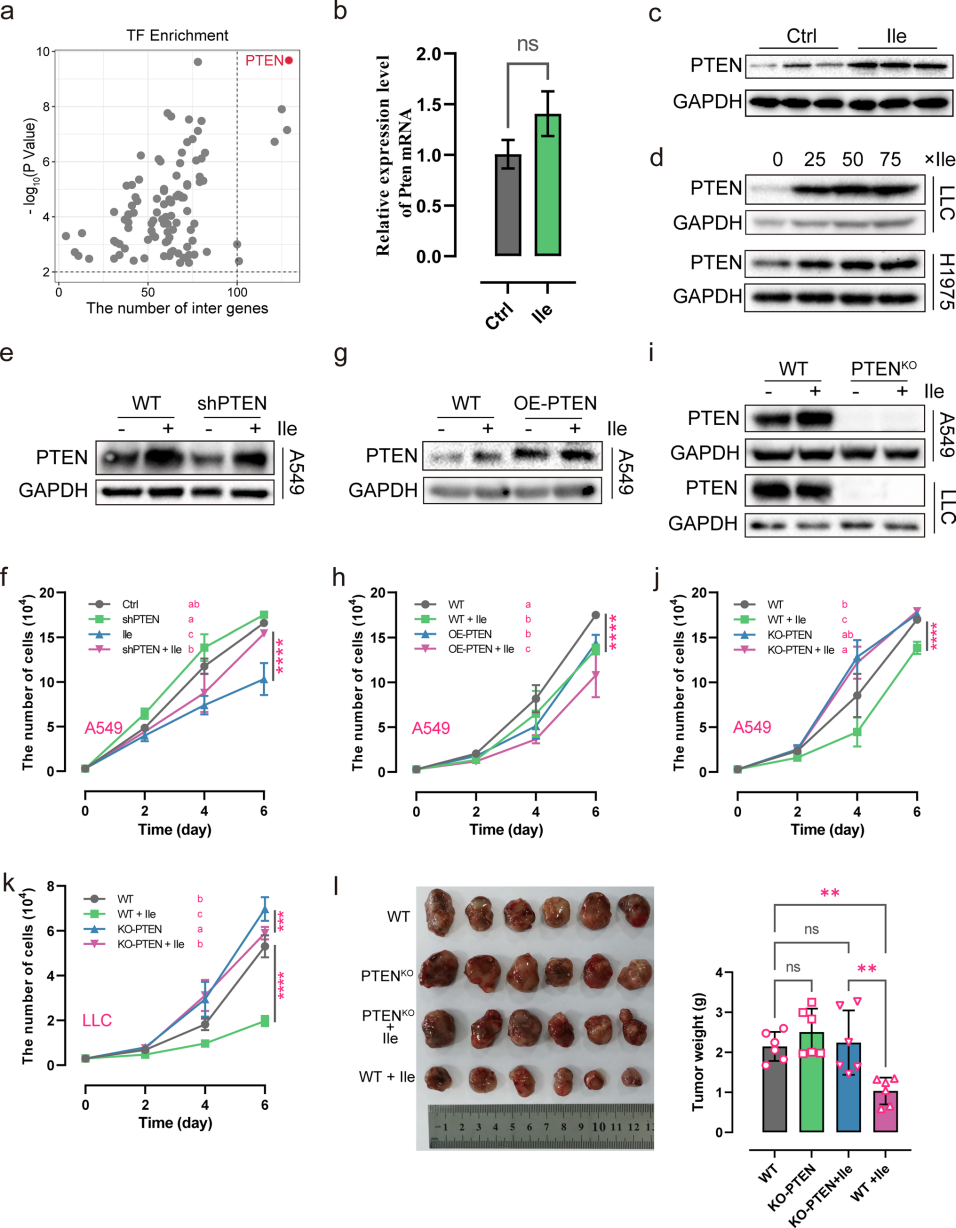
The anti-tumor activity of isoleucine is dependent on PTEN. **a** The scatter plot of enriched transcription factors for differentially expressed genes; **b** The relative expression level of Pten mRNA in LLC tumor tissues (n = 3); **c** PTEN protein level in LLC tumor tissues (n = 3); **d** PTEN protein level in LLC cells and H1975 cells treated with different concentrations of isoleucine for 48 h (n = 3); **e**–**k** Growth curve (in vitro) (n = 6) and the protein level of PTEN (48 h) (n = 3)in A549^shPTEN^ cells **e**–**f**, A549^OE−Flag−PTEN^ cells **g**–**h**, A549^KO−PTEN^ cells **i**–**j**, LLC^KO−PTEN^ cells (**I** and **k**), A549^WT^ cells **e**–**j** and LLC^WT^ cells (**I** and **k**) treated with 50 × isoleucine; **l** Assessment (tumor images and tumor weight) of isoleucine (Ile) on tumor growth based on LLC^KO−PTEN^ and LLC^WT^ tumor-bearing mice (n = 6). *ns* no significant difference; * *p* < 0.05; ** *p* < 0.01; *** *p* < 0.001; **** *p* < 0.0001. Different letters, *p* < 0.05, same letters, no significant difference

To find the critical regulators of isoleucine-regulated DEGs, the KnockTF tool [28] was implemented to obtain enriched transcription factors. The results showed that the transcription factor PTEN was the most relevant regulator of these DEGs and could regulate nearly 66.84% of the DEGs (129 DEGs, Fig. 3a & Supplementary Table 3). In LLC tumor tissue, there was no significant change in PTEN mRNA levels, but an increase in protein levels (Fig. 3b–c). In contrast, the Western blot results showed that PTEN protein levels in LLC and H1975 cells increased under isoleucine treatment (Fig. 3d). This phenomenon demonstrated that isoleucine may influence the post-translational level of PTEN, rather than the transcriptional level, to achieve its anti-tumor activity.

To further confirm the role of PTEN in isoleucine anti-tumor effects, we constructed a PTEN knockdown A549 cell line. In PTEN knockdown cells, the PTEN protein level was down-regulated compared to wild-type cells (Fig. 3e). However, its protein level was restored under isoleucine treatment (Fig. 3e). Knockdown of PTEN had a particularly proliferative effect on A549 cells compared to wild-type cells (Fig. 3f). The inhibitory effect of isoleucine was attenuated in A549 cells with PTEN knockdown (Fig. 3f). Similarly, stable overexpression of PTEN can inhibit the proliferation of A549 cells (Fig. 3g–h). At the same time, isoleucine can enhance this inhibitory effect (Fig. 3g–h). These results implied that isoleucine exerts its tumor suppressor effect by increasing the protein level of PTEN.

Next, we constructed PTEN-knockout A549 and LLC cell lines to investigate the isoleucine-PTEN-anti-tumor axis (Fig. 3i). Furthermore, the results showed that PTEN knockdown enhanced the cell proliferation ability. Significantly, the loss of PTEN abolished the anti-proliferative effect of isoleucine on A549 and LLC cells (Fig. 3j–k). To avoid bias caused by differences between in vitro experiments and normal physiological conditions, C57BL/6 mice were inoculated with PTEN knockout LLC cells and treated with isoleucine. Consistently, isoleucine reduced tumor weight in wild-type mice but lost its inhibitory effect on PTEN-deficient tumors (Fig. 3l). In summary, these proofs suggested that PTEN plays a crucial role in isoleucine-mediated tumor suppression.

### Isoleucine promotes nuclear entry of PTEN and maintains its stability

**Fig. 4:**
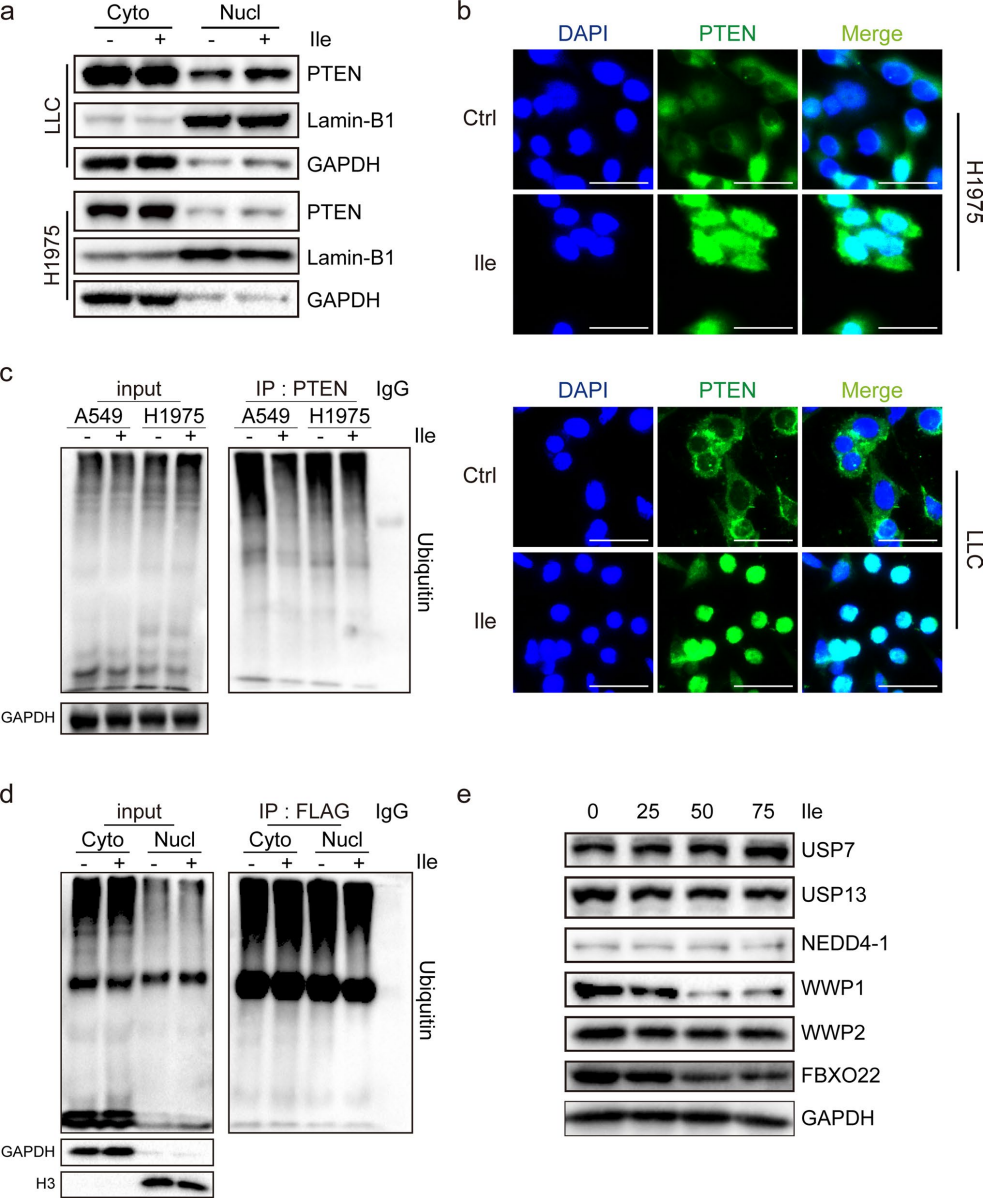
Isoleucine promotes the stability of nuclear PTEN based on the PTEN nuclear accumulation. **a** LLC cells and H1975 cells were treated with 50 × isoleucine for 48 h and subjected to Western blot analysis after extraction of cytoplasmic (Cyto) and nuclear (Nucl) proteins (n = 3); **b** PTEN immunofluorescent staining in H1975 cells and LLC cells, scale bar represents 50 μm (n = 3); **c** A549 cells and H1975 cells were treated with 50× isoleucine for 48 h and subjected to immunoprecipitation with an anti-PTEN antibody, followed by Western blot analysis with ubiquitination antibodies as indicated (n = 3); **d** Stably overexpressed Flag-PTEN A549 cells were treated with 50 × isoleucine for 48 h and cytoplasmic (Cyto) and nuclear (Nucl) proteins were immunoprecipitated with anti-Flag antibody followed by Western blot analysis using ubiquitination antibody (n = 3); **e** A549 cells were treated with different concentrations of isoleucine for 48 h, and related proteins involved in PTEN ubiquitination were analyzed by Western blot (n = 3)

As a transcription factor, PTEN needs to enter the nucleus to exert its regulatory activity. Hence, it is necessary to confirm whether isoleucine enhances the nuclear import of PTEN. As expected, nuclear PTEN protein levels were increased under isoleucine treatment, whereas cytoplasmic PTEN protein levels remained largely unaffected (Fig. 4a). Furthermore, immunofluorescence experiments displayed that PTEN proteins tended to accumulate in the nucleus in a high isoleucine environment (Fig. 4b). A critical mechanism for maintaining PTEN protein levels under physiological conditions is ubiquitin-mediated protein degradation, in which nuclear PTEN is more susceptible to it than cytoplasmic PTEN [34, 35]. Notably, we demonstrated that excessive supplementation of isoleucine directly leads to decreased PTEN ubiquitination (Fig. 4c). More importantly, isoleucine primarily reduces the ubiquitination of nuclear PTEN, but not cytoplasmic PTEN (Fig. 4d). At the same time, the detection of various ubiquitination-related proteins, including PTEN ubiquitination inhibitory proteins USP7 [36] and USP13 [37] and PTEN ubiquitination-promoting protein NEDD4-1 [38], WWP1 [34], WWP2 [39] and FBXO22 [35], showed that only WWP1 and FBXO22 were significantly down-regulated, while others were not significantly changed (Fig. 4e). These results suggest that isoleucine promotes the entry of PTEN into nuclear, and maintains the stability of nuclear PTEN by inhibiting the ubiquitination, thereby exerting its transcriptional regulation of tumor suppressor.

### Isoleucine-induced IARS mediates nuclear import of PTEN

**Fig. 5:**
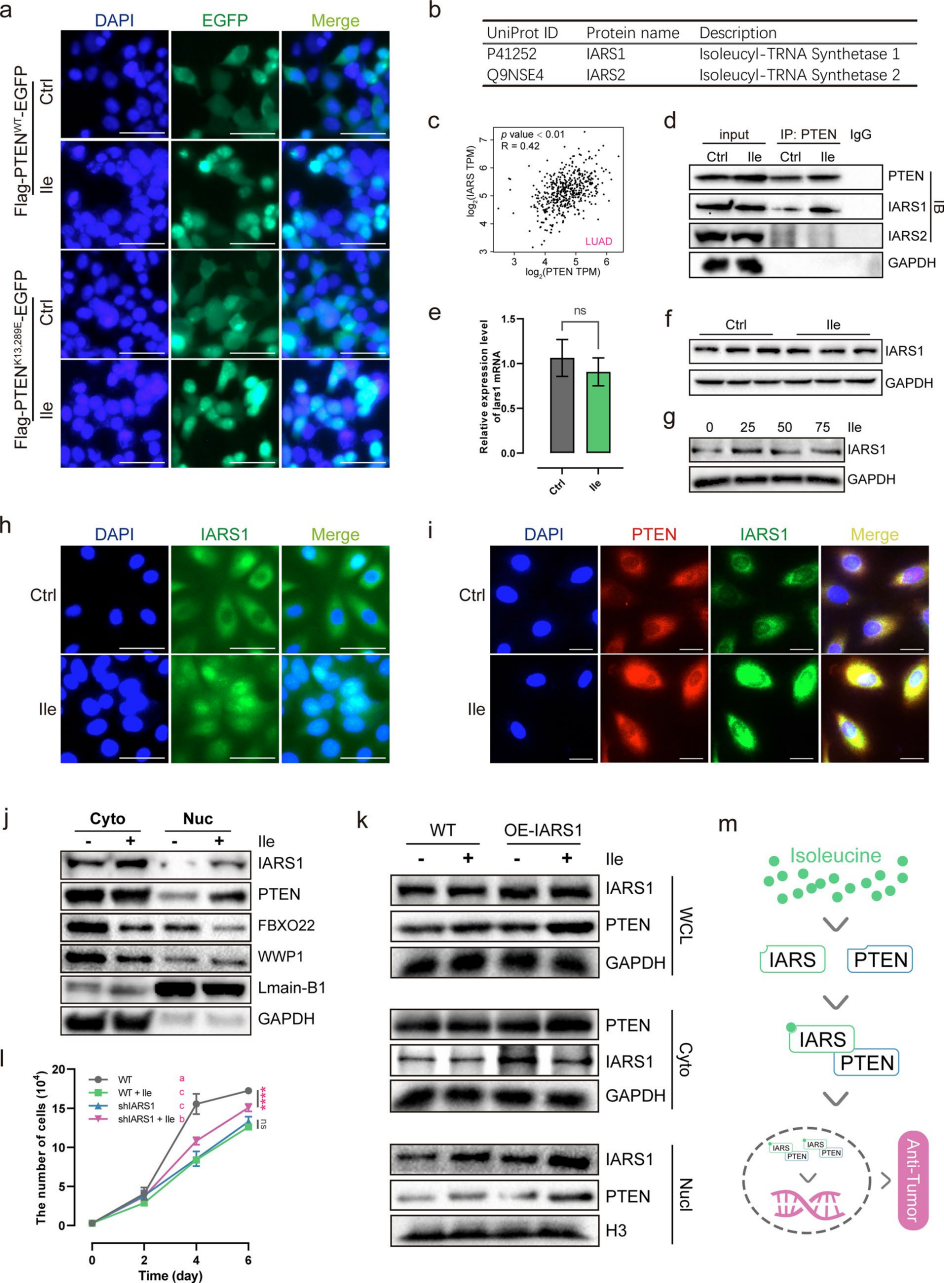
The rule between IARS and PTEN in the presence of isoleucine. **a** Immunofluorescent staining of 293 T cells transfected with plasmids expressing Flag-PTEN-EGFP or Flag-PTEN-EGFP^K13,289E^ for 4 h, and then treated with 50 × isoleucine for 12 h, scale bar represents 50 μm (n = 3); **b** Proteins most directly associated with isoleucine in proteins precipitated by anti-Flag-PTEN; **c** Pearson correlation between IARS1 and PTEN in LUAD from TCGA database; **d** A549 cells treated with 50 × isoleucine for 48 h were immunoprecipitated with anti-PTEN antibody and subjected to Western blot analysis. IgG was used as negative control (n = 3); **e** Relative expression level of Iars1 mRNA in LLC tumor tissues (n = 3); **f** IARS1 protein level in LLC tumor tissues (n = 3); **g** IARS1 protein level in H1975 cells treated with different concentrations of isoleucine for 48 h (n = 3); **h** IARS1 immunofluorescent staining in H1975 cells, scale bar represents 50 μm (n = 3); **i** Immunofluorescent colocalization between IARS1 and PTEN in A549 cells (n = 3); **j** A549 cells were treated with 50 × isoleucine for 48 h and subjected to Western blot analysis after extraction of cytoplasmic (Cyto) and nuclear (Nucl) proteins (n = 3); **k** Western blot analysis of cytoplasmic (Cyto), nuclear (Nucl), whole-cell lysates (WCL) in A549 cells transfected with plasmids expressing Flag-IARS1-EGFP for 4 h, and then treated with 50 × isoleucine for 12 h (n = 3); **l** Growth curve of stably IARS1-knockdown (IARS1) A549 cells and wild type (WT) A549 cells treated with 50 × isoleucine (n = 6); **m** The schematic diagram of the anticancer mechanism of isoleucine-mediated interaction between IARS and PTEN. *ns* no significant difference; *** *p* < 0.001. Different letters, *p* < 0.05, same letters, no significant difference. Different letters, p < 0.05, same letters, no significant difference

The above studies show altered ubiquitination of PTEN, and the monoubiquitination of PTEN at K13 and K289 is the primary mechanism regulating its nuclear entry [35, 40]. Hence, we transfected 293 T cells with plasmids expressing EGFP-tagged K13 and K289 mutated PTEN (PTEN^K13,289E^) and wild-type PTEN (PTEN^WT^) to detect the ubiquitous effect of isoleucine on K13 and K289 of PTEN. Surprisingly, fluorescence assays showed that isoleucine did not affect the nuclear localization of EGFP-tagged PTEN^K13,289E^ in the nuclei of 293 T cells (Fig. 5a). These data implied that there might be other mechanisms accounting for isoleucine-mediated entry of PTEN into the nucleus.

Hence, anti-Flag magnetic beads were used to precipitate PTEN-interacting proteins in A549 cells stably expressing Flag-PTEN. We obtained 1007 potential interacting proteins using liquid chromatography-tandem mass spectrometry (LC-MS/MS) analysis (Supplementary Table 4). Interestingly, among these candidate proteins, Isoleucine-tRNA ligase, cytoplasmic (IARS, also known as IARS1) and Isoleucine-tRNA ligase, mitochondrial (IARS2) are the proteins most directly related to isoleucine (Fig. 5b). Analysis of sequencing data from clinical NSCLC samples in the TCGA database showed that the expression of IARS1 (R = 0.42, *p* < 0.01) and IARS2 (R = 0.38, *p* < 0.01) both showed a potential positive correlation with PTEN expression (Fig. 5c & Supplementary Fig. 3a–c). Subsequently, co-immunoprecipitation results also verified that both ARS1 and IARS2 interact with PTEN (Fig. 5d). IARS1 showed a strong interaction with PTEN. This interaction was further enhanced by isoleucine. In contrast, the interaction between IARS2 and PTEN was much weaker, and unaffected by isoleucine (Fig. 5d). These results suggest that the isoleucine-enhanced interaction between IARS1 and PTEN may contribute to the nuclear import of PTEN and play a key role in isoleucine anti-tumor effects.

To confirm the potential role of IARS1 in isoleucine-treated tumors, we further probed for changes in IARS1 expression levels. Interestingly, isoleucine did not affect the mRNA and protein levels of IARS1 (Fig. 5e–f). Similarly, increasing the concentration of isoleucine failed to elicit a significant shift in IARS1 protein levels in cells (Fig. 5g). However, IARS1 subcellular localization analysis by MultiLoc2 tool [41] showed that 95% of IARS1 localized in the cytoplasm, 5% in the nucleus, and 1% in the mitochondria (https://abi-services.cs.uni-tuebingen.de/multiloc2/webloc.cgi, P41252). Moreover, immunofluorescence experiments also confirmed the accumulation of IARS1 in the nucleus under high isoleucine concentration (Fig. 5h). Immunofluorescence colocalization showed that IARS1 and PTEN co-aggregate near the nucleus under the influence of isoleucine (Fig. 5i). In addition, IARS1 protein levels in the nucleus were significantly increased by isoleucine (Fig. 5j). Further detection of nuclear and cytoplasmic ubiquitinated proteins revealed that WWP1 was reduced only in the cytoplasm, and FBXO22 was significantly reduced in both the cytoplasm and nucleus (Fig. 5j). Meanwhile, overexpression of IARS1 did not affect the protein level of nuclear PTEN, and only isoleucine significantly increased nuclear PTEN levels, consistent with previous results (Fig. 5k). Consistently, the knockdown of IARS1 inhibited the proliferation of A549 cells, resulting in the loss of the proliferation inhibition of A549 cells by isoleucine (Fig. 5l). These data suggest a dual role for IARS1. Usually, IARS1 is an essential element for maintaining cell proliferation. However, in excess isoleucine, the interaction between IARS1 and PTEN is enhanced and promotes its accumulation to the nucleus, thereby exerting PTEN transcriptional regulation of anti-tumor effects (Fig. 5m).

## Discussion

Malignant proliferation is one of the essential characteristics of tumor cells [42], and amino acids are an important energy source for tumor development. Over the past few decades, researchers have revealed that changes in signaling pathways such as transport systems, gene silencing, and redox homeostasis in the tumor microenvironment maintain an adequate supply of amino acids for tumors to achieve the drivers required for their proliferation [1]. However, amino acid deprivation or excess supply can disrupt the amino acid balance, thereby affecting the proliferation of cancer cells [2, 4, 5]. Hence, new therapies were proposed to perturb the balance of amino acids that might block tumor growth in the tumor microenvironment [43, 44]. Although the role of amino acids in cancer, especially essential amino acids, including branched-chain amino acids, has been intensively investigated in recent years [11, 12], supplementation or deprivation of amino acids remains a clinical challenge with limited preclinical rationale.

Accidentally, we found that high BCAA nutrition slowed the growth of various cancer cells in vitro, including NSCLC A549 & H1975), breast cancer (4T1), liver cancer (Hepa1-6), colon cancer (CT26) and clear cell renal cell carcinoma (786-O). Furthermore, in the mouse tumor xenograft model, excessive BCAA supplementation significantly inhibited the growth of NSCLC, colon cancer, and breast cancer but had no significant effect on melanoma. Paradoxically, high dietary BCAA intake or BCAA depletion promotes tumor initiation and growth [32, 45, 46]. Leucine is thought to be an important nutrient that is a promoter of tumor formation and development [16]. Further investigation of the anti-tumor activity of the three amino acids in BCAA also confirmed that only excess isoleucine or valine supplementation inhibited tumor growth in LLC tumor-bearing mice, with the inhibitory effect of isoleucine being most pronounced. These findings suggest that BCAA supplementation may improve the survival of cancer patients, especially isoleucine supplementation as a necessary adjuvant therapy for NSCLC patients. However, it should be noted that excessive intake and accumulation of BCAA in the blood is not beneficial to MSUD patients, but will induce acute severe ketoacidosis and aggravate a number of neurological diseases (apnea, epilepsy, mental retardation, etc.) [47, 48]. Therefore, the specific status of the patient must be considered in the clinical use of adjuvant BCAA therapy.

Interestingly, further research found that excess isoleucine activated mTORC1, a sensor for nutrients such as amino acids. Theoretically, activating this pathway promotes carcinogenesis but contradicts our observed anti-tumor activity of excess isoleucine. In the follow-up exploration, we found that excess isoleucine affects the interaction between IARS1 and the transcription factor PTEN, induces the entry of PTEN into the nucleus, and maintains its high protein level by reducing the ubiquitination of PTEN in the nucleus, and finally exerts its anti-tumor effect. The present work demonstrates that endogenous small molecules in the process of tumorigenesis, including nutritional molecules such as isoleucine, participate in the metabolic reprogramming process of tumors to meet the needs of tumor proliferation. [49]. On the other hand, we believe that once the supply balance of nutrient molecules is broken, such as excessive supplementation of isoleucine, endogenous nutrient molecules can also play a "drug"-like role, regulating the signaling pathways of the tumor microenvironment, thereby exerting the anti-tumor effect.

In conclusion, this study identifies an anti-tumor axis of excess isoleucine-IARS1-PTEN, which provides a new theoretical basis for amino acid supplementation in treating tumors. It should be noted that the interaction between IARS1 and PTEN in this paper still needs to be further determined by high-resolution confocal microscopy to determine the IARS1- PTEN interaction in the cell substructure (nucleus, cytoplasm). At the same time, IARS2 is also an important factor influencing the interaction between IARS1 and PTEN. Furthermore, this mechanism is only one of the potential anti-tumor mechanisms of isoleucine. The exploration of other anti-tumor mechanisms of isoleucine continues to be a focus of our research team. For example, isoleucine can also prevent liver metastases from colon cancer by down-regulating angiogenesis through the mTORC1-VEGF axis [50]. However, amino acid supplementation or deprivation can affect distinct tumors and patient prognosis at pathological stages of the same tumor differently [5, 51]. Moreover, there still needs to be broad consensus on the impact of the disruption of the supply and demand balance of amino acids in the tumor microenvironment on the occurrence, development, and spread of tumors. Next, multi-dimensional mechanism exploration would be valuable in targeting isoleucine. At the same time, the analysis of isoleucine from the perspective of metabolic recombination will also provide a comprehensive cognitive map for its anti-tumor mechanism. In addition, the contrasting study of isoleucine deprivation and supplementation may provide a more comprehensive understanding of the effect of isoleucine intake on tumors and provide a reference for its clinical application. Based on this demand, new research strategies and models are urgently needed to understand the impact of amino acid supply and demand balance on the tumor microenvironment. Multi-level research on amino acid anti-tumor atlas from the perspectives of metabolic reprogramming, signal regulation, and multi-tissue linkage will provide data support and a theoretical basis for amino acid clinical anti-tumor therapy.

## Supplementary Information

**Additional file 1. Supplementary Figure 1:** Sequencing validation of CRISPR/cas9 mediated PTEN knockout

**Additional file 2. Supplementary Figure 2:** Isoleucine classic pathway verification and GO enrichment analysis. (a) Growth curve of H1975 cell treated with rapamycin (50 μM), isoleucine (50×), and their combination in vitro; (b) S6K phosphorylation level in H1975 cells treated with rapamycin (50 μM), isoleucine (50×), and their combination in vitro; (c) Heatmap of differentially expressed genes; (d) GOBP enrichment analysis of differentially expressed genes was performed using Metascape. *ns* no significant difference; **** *p* < 0.0001. Different letters, *p* < 0.05, same letters, no significant difference.

**Additional file 3. Supplementary Figure 3:** The Pearson correlation between IARS1/IARS 2 and PTEN in LUAD and LUSC from the TCGA database.

**Additional file 4: Table S1.** 193 Differentially Expressed Genes

**Additional file 5: Table S2.** Gene Ontology Biological Process (GOBP) enrichment analysis based on ClueGO

**Additional file 6: Table S3.** Enriched transcription factors based on differentially expressed genes.

**Additional file 7: Table S4.** 1007 PTEN potential interacting proteins using liquid chromatography-tandem mass spectrometry (LC-MS/MS) analysis.

## Data Availability

The datasets generated during and/or analyzed during the current study are available from the corresponding author on reasonable request.
