# Supplementary figures and images for: High dose isoleucine stabilizes nuclear PTEN to suppress the proliferation of lung cancer
Source: Discov Oncol. 2023 Feb 23;14:25. doi: 10.1007/s12672-023-00634-1 (PMC9950318; doi:10.1007/s12672-023-00634-1)

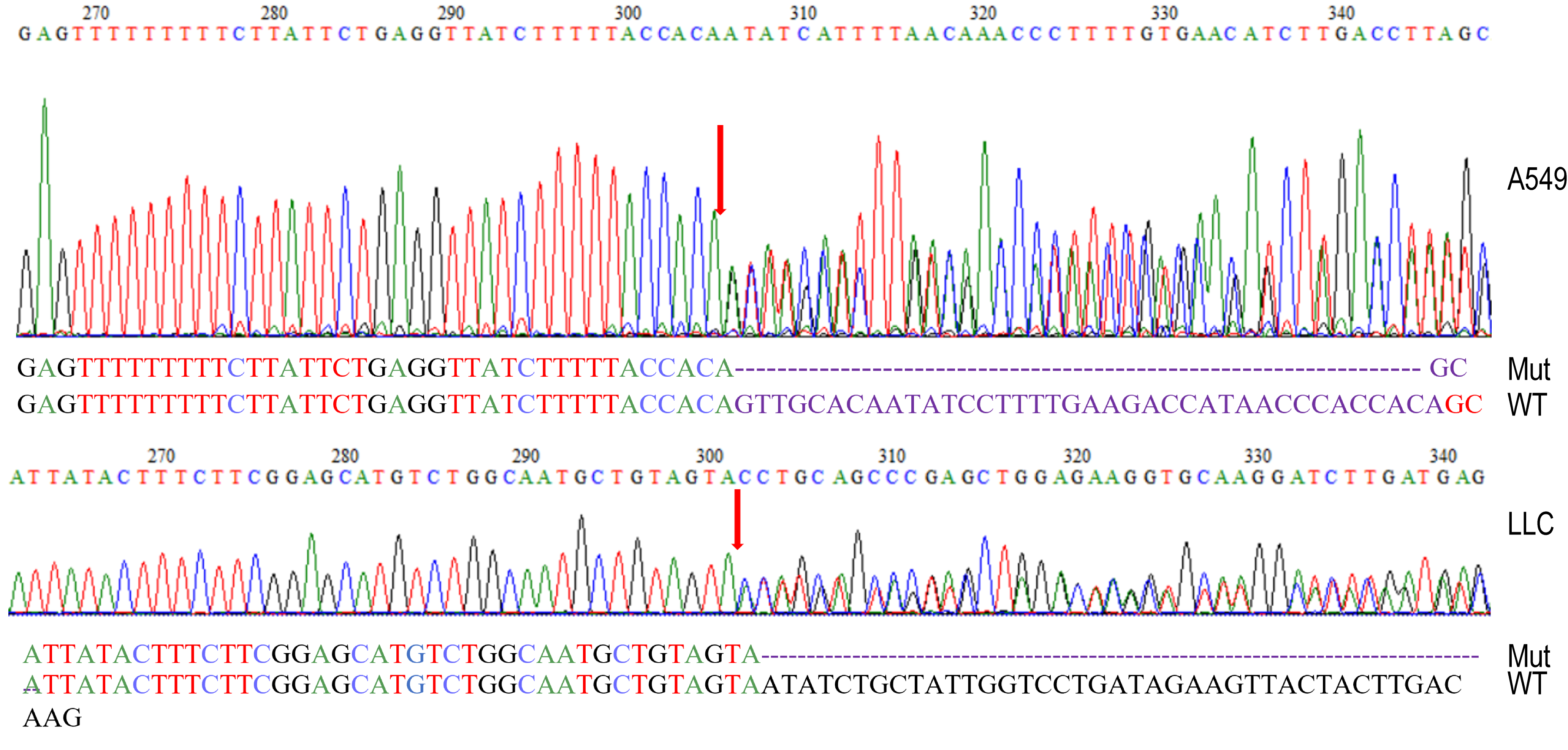

Supplement: Supplementary file 1 — Additional file 1. Supplementary Figure 1: Sequencing validation of CRISPR/cas9 mediated PTEN knockout [file 12672_2023_634_MOESM1_ESM.tif]

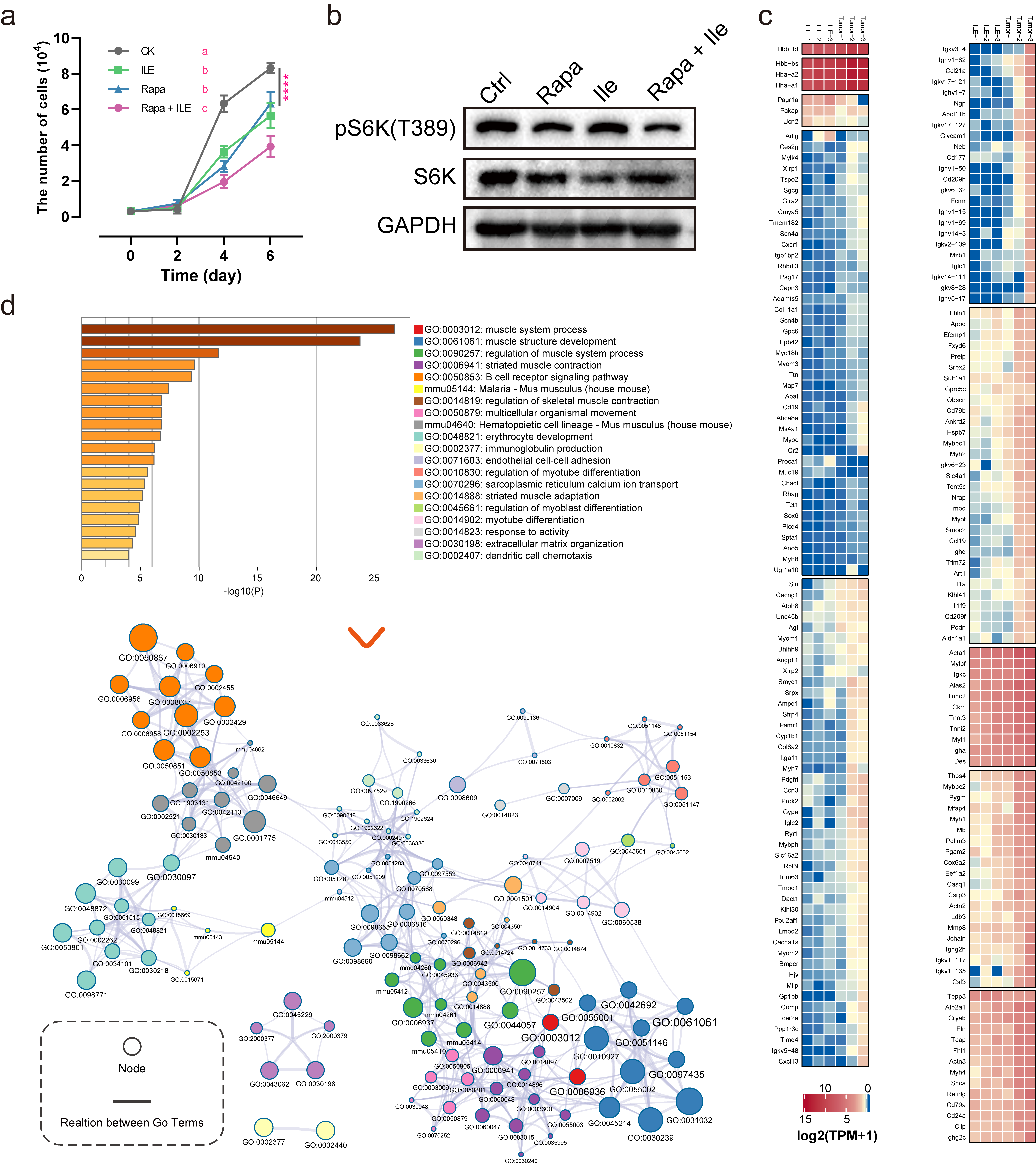

Supplement: Supplementary file 2 — Additional file 2. Supplementary Figure 2: Isoleucine classic pathway verification and GO enrichment analysis. (a) Growth curve of H1975 cell treated with rapamycin (50 μM), isoleucine (50×), and their combination in vitro; (b) S6K phosphorylation level in H1975 cells treated with rapamycin (50 μM), isoleucine (50×), and their combination in vitro; (c) Heatmap of differentially expressed genes; (d) GOBP enrichment analysis of differentially expressed genes was performed using Metascape. ns no significant difference; **** p < 0.0001. Different letters, p < 0.05, same letters, no significant difference. [file 12672_2023_634_MOESM2_ESM.tif]

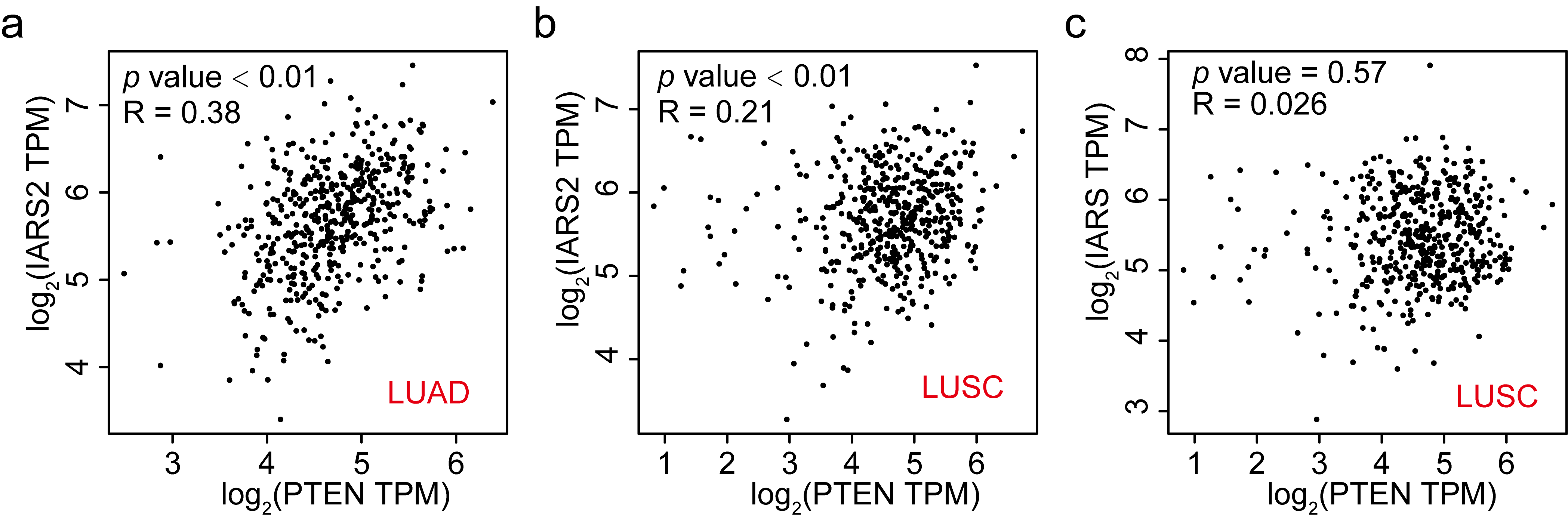

Supplement: Supplementary file 3 — Additional file 3. Supplementary Figure 3: The Pearson correlation between IARS1/IARS 2 and PTEN in LUAD and LUSC from the TCGA database. [file 12672_2023_634_MOESM3_ESM.tif]
